# Bone mass and quality in patients with juvenile idiopathic arthritis: longitudinal evaluation of bone-mass determinants by using dual-energy x-ray absorptiometry, peripheral quantitative computed tomography, and quantitative ultrasonography

**DOI:** 10.1186/ar4525

**Published:** 2014-03-31

**Authors:** Stefano Stagi, Loredana Cavalli, Carla Signorini, Federico Bertini, Marco Matucci Cerinic, Maria Luisa Brandi, Fernanda Falcini

**Affiliations:** 1 Health’s Sciences Department, University of Florence, Florence, Italy; 2 Department of Internal Medicine, Endocrinology Unit, University of Florence, Florence, Italy; 3 Department of Internal Medicine, Section of Rheumatology, Transition Clinic, University of Florence, Viale Pieraccini 18, 50139 Florence, Italy

## Abstract

**Introduction:**

Our objective was to evaluate longitudinally the main bone-mass and quality predictors in young juvenile idiopathic arthritis (JIA) patients by using lumbar spine dual-energy X-ray absorptiometry (DXA) scan, radius peripheral quantitative computed tomography (pQCT), and phalangeal quantitative ultrasonography (QUS) at the same time.

**Methods:**

In total, 245 patients (172 females, 73 males; median age, 15.6 years: 148 oligoarticular, 55 polyarticular, 20 systemic, and 22 enthesitis-related-arthritis (ERA) onset) entered the study. Of these, 166 patients were evaluated longitudinally. Data were compared with two age- and sex-matched control groups.

**Results:**

In comparison with controls, JIA patients, but not with ERA, had a reduced spine bone-mineral apparent density (BMAD) standard deviation score (*P* < 0.001) and musculoskeletal deficits, with significantly lower levels of trabecular bone mineral density (TrabBMD) (*P* < 0.0001), muscle cross-sectional area (CSA) (*P* < 0.005), and density-weighted polar section modulus (SSIp) (*P* < 0.05). In contrast, JIA showed fat CSA significantly higher than controls (*P* < 0.0001). Finally, JIA patients had a significant reduced amplitude-dependent speed of sound (AD-SoS) (*P* < 0.001), and QUS *z* score (*P* < 0.005).

Longitudinally, we did not find any difference in all JIA patients in comparison with baseline, except for the SSIp value that normalized. Analyzing the treatments, a significant negative correlation among spine BMAD values, TrabBMD, AD-SoS, and systemic and/or intraarticular corticosteroids, and a positive correlation among TNF-α-blocking agents and spine BMAD, TrabBMD, and AD-SoS were observed.

**Conclusions:**

JIA patients have a low bone mass that, after a first increase due to the therapy, does not reach the normal condition over time. The pronounced bone deficits in JIA are greater than would be expected because of reduction in muscle cross-sectional area. Thus, bone alterations in JIA likely represent a mixed defect of bone accrual and lower muscle forces.

## Introduction

In juvenile idiopathic arthritis (JIA), a multifactorial reduction of bone mineral accrual with impaired bone mass has been widely reported [1-3]. Chronic inflammation [4-6], delayed pubertal maturation [3], malnutrition [7], muscle weakness [6], limited physical activity [8,9], early onset of JIA [3], and treatments, especially corticosteroids [1,10], influence negatively bone mineral density (BMD) in these subjects. Therefore, although the heredity accounts for a large proportion of variation in BMD, these factors may exert a significant negative influence on the development of peak bone mass (PBM) [3,11,12], with increased fracture risk in adulthood [13,14]. In addition, JIA patients, after a therapy-induced increase of BMD, do not seem to reach a normal condition over time [3].

To assess bone health appropriately, integration between clinical tools and imaging techniques is required [1,2]. Dual-energy x-ray absorptiometry (DXA) bone measures are based on a two-dimensional image that precludes a direct measure of volumetric BMD (vBMD). Because BMD only partly explains bone quality, these abnormalities may be better estimated by a quantitative assessment of macro- and microstructural characteristics [15]. Thus, three-dimensional imaging methods, such as peripheral quantitative computed tomography (pQCT), allow a selective assessment of both trabecular and cortical bone and also estimate bone strength [16]. In addition, quantitative ultrasonography (QUS), increasingly used for its low cost, portability, and lack of exposure to ionizing radiation, is used to evaluate bone density and structure and also permits assessment of changes in trabecular and cortical architecture [16].

Although several studies demonstrated reduced bone mass or quality in JIA patients [3,6,11,17-21], few large cross-sectional or prospective, case–control data exist for children and young adults [3,8,22] and few, in particular, using DXA, pQCT, and QUS scans simultaneously. Thus, the aim of the study was to evaluate bone mass and quality in children, adolescents, and young adults with JIA.

## Methods

Patients were recruited consecutively from the Rheumatology Unit of Florence University between January 2008 and July 2011. All underwent forearm pQCT, QUS scans at the phalanges, and DXA at the lumbar spine on the same day.

The study protocol was approved by Hospital Ethics Committee of Careggi University Hospital of Florence, and written informed consent was obtained from all patients and controls or their parents or guardians, as appropriate.

### Study population and design

Two hundred forty-five patients (172 female and 73 male patients; median age at study entry, 15.6; range, 9.6 to 28.3 years) fulfilling the criteria for JIA [23] were evaluated. One hundred forty-eight had oligoarticular; 55, polyarticular (rheumatoid factor negative); 20, systemic; and 22, enthesitis-related arthritis (ERA) onset. Of these, 166 patients (132 females, 34 males; 99 oligoarticular, 30 polyarticular, 18 systemic, and 19 ERA) were followed up longitudinally with a second DXA, pQCT, and QUS. Of the oligoarticular patients, 26% had extended arthritis at the first DXA evaluation, whereas an additional 13% were so classified at the second evaluation.

For each patient, clinical and demographic data, including JIA-type onset, age at diagnosis, disease duration, disease course, flares, therapy, height, pubertal stage, weight and body mass index (BMI), and family history of osteoporosis, were recorded from medical charts.

Onset of disease was defined as the date on which arthritis and/or systemic features were documented by a pediatric rheumatologist [3]. The disease subtype and the active JIA assessment were defined according to a previously reported definition [3,23]. Clinical assessment and laboratory evaluation were performed longitudinally to report the disease course and flares [3].

Exclusion criteria for both JIA patients and controls were bone metabolic diseases, hyper-/hypoparathyroidism, hyper-/hypothyroidism, chronic renal failure, cancer, pregnancy, lactation, and osteopenia-inducing drugs, on the basis of the medical history and questionnaires for osteoporosis risk factors.

### Study and laboratory methods

Height was measured by using a wall-mounted stadiometer, and weight was measured to the nearest 0.1 kg. Age-related reference values of height and BMI (kg/m^2^) were obtained from a wide sample of Italian children [24]. Height and BMI were normalized for chronologic age by conversion to standard deviation scores (SDSs).

Pubertal staging was carried out according to the Tanner and Whitehouse criteria [25], and testicular volume was determined with the Prader orchidometer. The timing of puberty and the comparison between the normal population and JIA patients were assessed [3].

The age at pubertal onset was defined as the age at durable Tanner B2 stage for girls and a testicular volume of more than 4 ml for boys (G2). The age at which the onset of puberty occurred was recorded as the average age between the previous clinic visit, when the child was still prepubertal, and the clinic visit when the child was G2/B2. The duration of puberty was recorded as the time from G2/B2 to G4/B4. The age at G4/B4 was assessed by averaging the ages at the previous clinic visit, when the child was G3/B3, and the clinic visit when the child was G4/B4.

### DXA scans

BMD was measured at the lumbar spine by dual-energy x-ray absorptiometry by using the same instrument (Delphi-A 4500 System; Hologic, Inc., Waltham, MA, USA). All BMD results were expressed in terms of g/cm^2^ or BMD *Z* scores. Average BMD values for L2 to L4 were used for calculations.

Because the DXA instrument’s software does not take into account the actual bone volume strictly related to body size (weight and height), estimation of the respective volumetric density (that is, bone mineral apparent density or BMAD) was determined by using the formula of Kröger *et al.*[26], as previously reported [3]:

$$\mathrm{BMAD}={\mathrm{BMD}}_{L2‒L4}\times \left(4/\left(\pi \times \mathrm{width}\right)\right),\mathrm{expressed}\;\mathrm{in}\;g/{\mathrm{cm}}^{3}.$$

The Kröger model was validated by using *in vivo* volumetric data obtained from magnetic resonance imaging of the lumbar vertebrae [27]. The intraobserver coefficient of variation was 1.0%.

Patients’ BMAD was expressed as Z scores (that is, the difference between the value of the patient and the normal value for age divided for standard deviation (SD) of the normal patients group) to facilitate comparisons between JIA patients and healthy controls.

### Peripheral quantitative computed tomography scan

The left (nondominant) radius was measured by using pQCT by a single, trained investigator, with a Norland-Stratec XCT 3000 scanner (Stratec Medical, Pforzheim, Germany).

Tomographic slices of 2-mm thickness with voxel sizes of 0.4 mm and a scan speed of 25 mm/sec were taken from the shaft and the distal part of the radius (66% and 4% from the distal end plate of the radius, respectively) [28].

Names for end-point variables were chosen according to the Task Force on Standardization of Bone Structure and Density Assessment [29] for high-resolution pQCT.

The 4% (distal) radial site allowed assessment of the trabecular bone to obtain total bone mineral density (TotBMD, mg/cm^3^) and trabecular bone mineral density (TrbBMD, mg/cm^3^) [28].

For the shaft regions (66%), the analyzed bone traits were cortical bone mineral density (CrtBMD, mg/cm^3^), cortical bone area (CBA, mm^2^), muscle cross-sectional area (MuscleCSA, mm^2^), fat cross-sectional area (FatCSA, cm^2^), and density-weighted polar section modulus (SSIp, mm^3^, an estimate of bending and torsional bone strength for cortical bone).

Because growth retardation is common in some JIA children and adolescents [1,2], all bone size-dependent parameters (Total, Cortical, and MuscleCSA) were corrected for height.

### QUS scans

QUS scans were performed with a DBM Sonic 1200 device (IGEA Bone Profiler, Carpi, Italy), equipped with two probes mounted on an electronic caliper. With this technique, we measured (1) amplitude-dependent speed of sound (AD-SoS, in m/s), the interval between the start time of the transmitted signal and the time the signal received reaches the predetermined minimum amplitude value of 2 mV for the first time); and (b) bone transmission time (BTT, in μsec), the difference between transmission time in phalanx soft tissue and bone and transmission time in phalanx soft tissue. AD-SoS and BTT SDS were automatically generated [16].

### Clinical assessment of disease activity

Clinical disease activity was determined as previously described [3]. Documentation of patient data included medical history and physical examination, in particular the number of active joints, number of joints with limited range of motion, physician’s global assessment of disease activity, parent’s/patient’s assessment of overall well-being (visual analogue scale as part of the Childhood Health Assessment Questionnaire [C-HAQ]), and functional ability (disability as measured in eight domains by the C-HAQ). Patients were categorized as having active disease or being in clinical remission for at least 6 consecutive months, as previously described [3]. Relapse was defined according to the preliminary definition of disease flare in JIA [3,29].

### Type of treatment

During the cross-sectional and longitudinal evaluations, the following therapies were considered to define the task of these determinants on spine BMAD values: nonsteroidal antiinflammatory drugs (NSAIDs), sulfasalazine, systemic and intraarticular corticosteroids, methotrexate (MTX), and TNF-α-blocking agents [3]*.*

For all drugs, the period of administration was also obtained from the outpatient clinic and hospital records at the time of the first and second evaluations. Moreover, the number of intraarticular glucocorticoids injections and the interval between the injection and DXA assessment were noted. Systemic corticosteroid dosages were converted into a common steroid equivalency (mg/kg body weight per day of prednisolone-equivalent dose).

### Healthy control subjects

At the first evaluation, the control group included 80 healthy age- and sex-matched subjects (58 females, 22 males; median age, 14.8 years; range, 8.9 to 26.7 years), observed at our medical units for noninflammatory musculoskeletal complaints. At the time of the second scans, we selected another age- and sex-matched control group with the same characteristics (82 subjects, 59 females, 23 males; median age, 16.9 years; range, 10.9 to 28.4 years). The data for part of these groups were also previously reported [3].

### Statistical analysis

Statistical analyses were performed by using SPSSX (SPSSX Inc., Chicago, IL, USA). Summaries of continuous variables were given as the means ± SDs or median and range, depending on whether the data were normally distributed. Differences between patient groups and controls were assessed by using the Student *t* test and Mann–Whitney *U* test, depending on the distribution of the analyzed variable. The χ^2^ test and the Fisher Exact test were used as appropriate to examine associations between dichotomous variables. Intergroup comparisons for parameters were conducted by using analysis of variance (ANOVA) or by using repeated-measures analysis of covariance (ANCOVA), as appropriate.

Spearman and Pearson correlation tests were used to determine the correlation coefficients. A multiple stepwise regression was used to determine the variables that correlated independently with lumbar spine BMAD, QUS, and pQCT *z* scores. *P* values <0.05 were considered statistically significant.

## Results

The general data of the study population and controls are shown in Table 1.

**Table 1:** Data for the 141 JIA patients longitudinally evaluated in comparison to their respective control groups

	**JIA group**		**Control group**	
	**Baseline**	**Second evaluation**	**Baseline**	**Second evaluation**
Number of subjects	245	166	80	82
Female/male ratio	172:73	132:34	58:22	59:23
Age, years (range)	15.6 (9.6 - 28.3)	17.1 (11.0 - 28.9)	14.8 (8.9 - 26.7)	16.9 (10.9 - 28.4)
Height, *z* score	−0.6 ± 1.1	−0.5 ± 0.9	−0.1 ± 0.9	−0.1 ± 0.7
BMI, *z* score	−0.3 ± 0.8	−0.1 ± 0.6	0.1 ± 0.7	0.2 ± 0.5
Disease duration	5.6 ± 3.2 years	7.2 ± 4.3 years	-	-
C-HAQ DI	0.74 ± 0.63	0.59 ± 0.48	-	-
DXA examination				
BMC, g	45.17 ± 19.13^a^	49.04 ± 21.56°°°^j^	51.23 ± 17.90	56.98 ± 16.82^h^
BMAD spine, g/cm^2^	−0.64 ± 0.80^b^	−0.49 ± 1.10°°°	0.24 ± 0.21	0.13 ± 0.32
HR-pQCT				
TrbBMD (mg/cm^3^)	179.5 ± 34.6^c^	181.9 ± 48.8^f^	217.7 ± 26.3	244.9 ± 28.5^i^
CBA (mm^2^)	136.3 ± 122. ^c^	149.0 ± 87.6^f^	209.2 ± 101.3	214.3 ± 74.3
CrtBMD (mg/cm^3^)	1,050.2 ± 126.5	1,079.8 ± 82.1^j^	1,039.6 ± 79.3	1,078.3 ± 73.1^g^
Muscle CSA (mm^2^)	2,112.7 ± 732.4^b^	2,083.7 ± 697.8^f^	2,398.5 ± 773.3	2,579.3 ± 794.5^h^
Fat CSA (mm^2^)	1,143.1 ± 649.1^c^	1,110.1 ± 483.7^e^	918.3 ± 382.1	945.8 ± 394.2
SSIp (mm^3^)	246.3 ± 140.2^a^	306.8 ± 250.7^d,j^	284.0 ± 127.9	276.7 ± 110.4
Ultrasonography				
-AD-SoS	1,994.5 ± 119.7^b^	2,003.0 ± 101.1^e^	2,122.3 ± 113.1	2,152.8 ± 118.9
-QUS *z* score	−0.6 ± 1.8^a^	−0.5 ± 1.5^e^	0.1 ± 1.6	0.2 ± 1.8

JIA versus controls (transversal study): ^a^*P* < 0.05; ^b^*P* < 0.005; ^c^*P* < 0.0005; JIA *versus.* controls (longitudinal study): ^d^*P* < 0.05; ^e^*P* < 0.005; ^f^*P* < 0.0005.

Controls *versus* controls (longitudinal study): ^g^*P* < 0.05; ^h^^^ *P* < 0.005; ^i^*P* < 0.0005; JIA *versus* JIA (longitudinal study): ^j^*P* < 0.05; ^§^j^§^*P* < 0.005; ^§§§^ < 0.0005.

AD-SoS, amplitude-dependent speed of sound; BMAD, bone mineral apparent density; BMC, bone mineral content; BMI, body mass index; CBA, cortical bone area; C-HAQ DI, Childhood Health Assessment Questionnaire Disability Index, range, 0–3 (0 = best, 3 = worst); CrtBMD, cortical bone-mineral density; DXA, dual-energy x-ray absorptiometry; FatCSA, fat cross-sectional area; HR-pQCT, high-resolution peripheral quantitative computed tomography; JIA, juvenile idiopathic arthritis; MuscleCSA, muscle cross-sectional area; SSIp, density-weighted polar section modulus; QUS, quantitative ultrasonography; TrbBMD, trabecular bone-mineral density.

With respect to exposure to different medications at study entry, 56% of the JIA patients had received NSAIDs. Methotrexate had been prescribed to 44.7% and TNF-α-blocking medications to 22% of patients; 30% had received sulfasalazine, and glucocorticoids were prescribed to 21.7%. High-dose systemic or oral glucocorticoids had been administered only to patients with systemic JIA onset (*P* < 0.001), whereas the dose administered in the other JIA subtypes was low, without significant differences among the different groups. Forty-one percent had taken glucocorticoids during the year before measurements.

With respect to disease activity, at study entry, 46.1% of the JIA patients had disease activity, whereas 53.9% of patients had remission/inactive disease. Our data related to the age at puberty onset, compared with controls, evidenced that age was delayed, but not significantly (years (95% CI)) both in girls (B2, 11.0 ± 2.0 versus 10.5 ± 1.5; B3, 11.8 ± 2.1 versus 11.1 ± 1.7; B4, 12.7 ± 1.9 versus 11.8 ± 1.6) than in boys (G2, 12.2 ± 2.4 versus 11.6 ± 1.8; G3, 13.4 ± 2.3 versu*s* 12.5 ± 2.1; G4, 14.8 ± 1.7 versus 13.6 ± 1.5).

**Figure 1:**
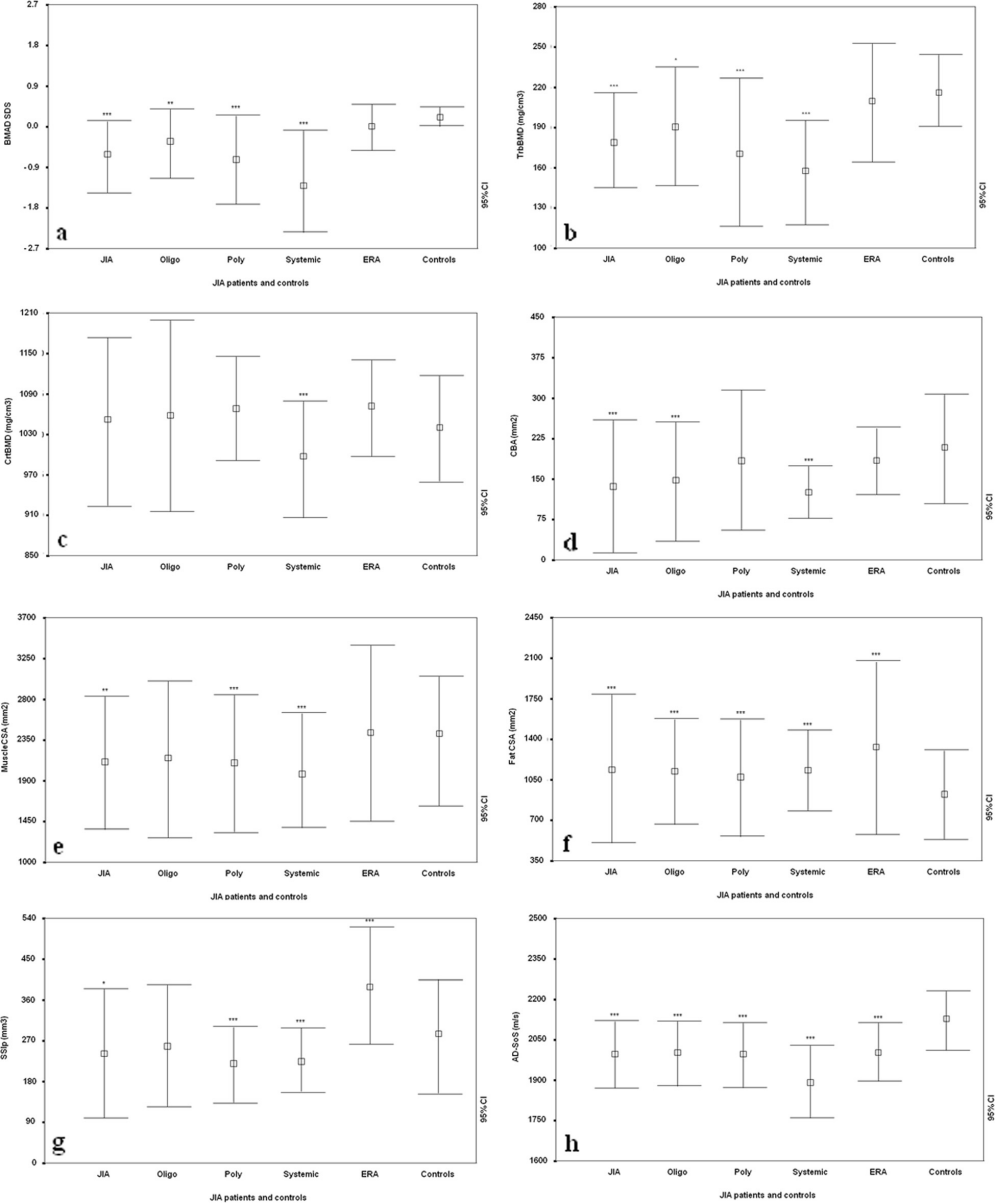
**Transversal evaluation.** Spine bone mineral apparent density (spine BMAD) SDS **(a)**, trabecular bone mineral density (TrabBMD) **(b)**, cortical bone mineral density (CrtBMD) **(c)**, cortical bone area ([CBA) **(d)**, muscle cross-sectional area (MuscleCSA) **(e)**, fat cross-sectional area (FatCSA) **(f)**, density-weighted polar section modulus (SSIp) **(g)**, and Ad-SoS **(h)** in patients with oligoarticular, polyarticular, systemic, and enthesitis-arthritis (ERA) JIA versus controls. Bars represent means and 95% confidence intervals. **P < 0.005; ***P < 0.001.

In the first evaluation, the JIA patients showed reduced spine BMAD SDS values in comparison with controls (−0.64 ± 0.80 versus 0.24 ± 0.21; *P* < 0.001); these results were also observed when the subjects were divided into oligoarticular (−0.36 ± 0.80; *P* < 0.005), polyarticular (−0.73 ± 1.01; *P* < 0.0001), and systemic (−1.34 ± 1.20; *P* < 0.0001) onset groups. Conversely, ERA subjects did not show any difference in comparison to controls (0.01 ± 0.52; *P* = NS) (Figure 1a).

In addition, significant musculoskeletal deficits were observed at the time of the first evaluation. The JIA patients had significantly lower levels of TrbBMD than did controls (179.5 ± 34.6 versus 217.7 ± 26.3 mg/cm^3^; *P* < 0.0001). These results were also observed when the subjects were divided into oligoarticular (191.9 ± 43.2, *P* < 0.05), polyarticular (172.5 ± 55.5, *P* < 0.0001), and systemic onset (157.6 ± 39.3, *P* < 0.0001) groups, but not in the ERA group (209.6 ± 44.4, *P* = NS) (Figure 1b).

In contrast, the JIA patients did not show significant differences in CrtBMD (Figure 1c) in comparison to the controls (1,050.2 ± 126.5 versus 1,039.6 ± 79.3, *P* = NS), even if we considered the different subtypes (oligoarticular, 1,057.5 ± 142.4; polyarticular, 1,068.1 ± 76.8; ERA, 1070.4 ± 71.2 mg/cm^3^), except systemic-onset patients, who had a significant reduction (994.0 ± 87.3 mg/cm^3^, *P* < 0.0001). At the same time, cortical bone area, CBA (Figure 1d), appeared significantly reduced in comparison to controls (136.3 ± 122.1 versus 209.2 ± 101.3 mm^2^; *P* < 0.0001), even if the subjects were divided into oligoarticular- (148.5 ± 112.0 mm^2^, *P* < 0.0001), and systemic-onset (126.3 ± 49.0 mm^2^, *P* < 0.0001) groups, but not when comparing the controls with the polyarticular (186.3 ± 131.1 mm^2^, *P* = NS) and ERA (187.5 ± 64.5 mm^2^, *P* = NS) groups.

The JIA patients also showed significantly lower muscle CSA (Figure 1e) than did controls (2,112.7 ± 732.4 versus 2,398.5 ± 773.3 mm^2^, *P* < 0.005). These differences relative to the controls were also confirmed in the polyarticular (2,100.5 ± 764.4 mm^2^, *P* < 0.001) and systemic-onset (2,023.2 ± 632.8 mm^2^, *P* < 0.0001) groups, but not in the oligoarticular (2,167.7 ± 870.2, *P* = NS) and ERA (2,420.2 ± 973.1 mm^2^, *P* = NS) groups.

Fat CSA (Figure 1f) was significantly increased in the JIA patients relative to the controls (1,143.1 ± 649.1 versus 918.3 ± 382.1 mm^2^, *P* < 0.0001), even when the patients were divided into the different subtypes (oligoarticular, 1,129.6 ± 451.0 mm^2^; polyarticular, 1,080.9 ± 504.8 mm^2^; systemic, 1,139.7 ± 351.9 mm^2^; ERA, 1,335.2 ± 748.2 mm^2^, *P* < 0.0001).

The JIA patients also had significantly lower SSIp levels (Figure 1g) than controls (246.3 ± 140.2 versus 284.0 ± 127.9 mm^3^, *P* < 0.05), even when divided into polyarticular (220.2 ± 82.9, *P* < 0.0001) and systemic-onset groups (228.0 ± 68.6, *P* < 0.0001), but not when controls were compared with the oligoarticular group (261.7 ± 134.5, *P* = NS). In contrast, subjects with ERA had significantly increased SSIp values with respect to controls (353.1 ± 129.9, *P* < 0.05) (Figure 1g).

Finally, in the JIA patients, AD-SoS (Figure 1h) results were significantly reduced (1,994.5 ± 119.7 versus 2,122.3 ± 113.1 m/s, *P* < 0.001), even when patients were divided into subgroups (oligoarticular: 2,004.7 ± 118.6 m/s; polyarticular: 1,992.2 ± 116.2 m/s; systemic: 1,894.3 ± 133.1 m/s; ERA: 2,003.4 ± 105.8 m/s, *P* < 0.0001). The results were comparable also when QUS *z*-scores in the JIA patients and controls were evaluated (−0.6 ± 1.8 versus 0.1 ± 1.6, *P* = 0.003) and between the subtypes and the controls (polyarticular: −1.0 ± 1.6; systemic: −2.7 ± 2.0; ERA: −0.8 ± 1.5, *P* < 0.0001), except for the oligoarticular-onset subtype (−0.4 ± 1.7, *P* = NS).

The correlations among the variables in the transversal study are reported in Tables 2 and 3.

**Table 2:** Correlation coefficients (rho) between the bone traits in the JIA cohort and controls

**pQCT phenotypes**								**DXA Phenotypes**			**QUS phenotypes**		
	**TrbBMD**	**CrtBMD**	**Total density**	**CBA**	**Bone area**	**Muscle CSA**	**Fat CSA**	**SSIp**	**BMAD**	**BMD**	**QUS z-score**	**AD-SoS**	**BTT**
TrbBMD		0.13	0.60***	0.10	0.14	0.26*	0.16	0.05	0.12	0.12	- 0.05	0.01	0.07
		0.19	**0.59^^^**	**0.23^**	0.19	**0.31^^**	**0.29^**	**0.28^**	**0.35^^**	0.15	0.09	0.14	0.18
		**0.25°**	**0.78°°°**	**0.32°°**	**0.24°**	**0.35°°**	−0.10	**−0.26°**	**0.39°°**	**0.44°°**	0.17	0.12	**0.31°**
CrtBMD			0.34**	0.23*	0.14	0.42***	0.09	0.36**	0.49**	0.69***	0.21*	0.60***	0.64***
			**0.50^^^**	**0.52^^^**	**0.28^**	**0.39^^**	0.16	**0.41^^**	**0.46^^**	**0.72^^^**	**0.22^**	**0.70^^^**	**0.66^^^**
			**0.39°°**	**0.83°°°**	**0.49°°**	**0.70°°°**	- **0.27°**	**0.39°°**	**0.63°°°**	**0.80°°°**	**0.61°°°**	**0.72°°°**	**0.82°°°**
Tot density				0.11	0.19	0.41**	0.23	0.22*	0.12	0.46**	0.27*	0.43***	0.40***
				**0.39^^**	0.15	**0.38^^**	−0.20	**0.23^**	**0.36^^**	**0.48^^**	**0.24^**	**0.50^^**	**0.42^^**
				**0.61°°°**	**0.29°**	**0.59°°°**	0.10	**0.32°°**	**0.24°**	**0.56°°°**	0.18	**0.35°°**	**0.43°°**
CBA					0.11	0.22*	- 0.26*	0.25*	- 0.09	0.28**	0.12	0.24*	0.25*
					**0.49^^**	**0.81^^^**	−0.16	**0.83^^^**	**0.25^**	**0.71^^^**	**0.31^^**	**0.48^^**	**0.72^^^**
					**0.82°°°**	**0.92°°°**	0.14	**0.42°°°**	**0.34°°**	**0.89°°°**	**0.57°°°**	**0.26°**	**0.85°°°**
Bone area						0.49**	0.05	0.55**	0.20	0.18	0.05	0.18	0.30**
						**0.49^^**	0.11	**0.66^^**	**0.31^^**	**0.40^^**	0.09	0.19	**0.37^^**
						**0.78°°°**	**0.37°°**	**0.72°°°**	**0.22°**	**0.75°°°**	**0.27°**	**0.47°°**	**0.71°°°**
MuscleCSA							- 0.27*	0.47***	0.53***	0.66***	0.15	0.48**	0.76***
							**−0.37^^**	**0.63^^^**	**0.42^^**	**0.61^^^**	0.13	**0.70^^^**	**0.80^^^**
							0.02	**0.38°°**	**0.34°°**	**0.86°°°**	0.19	**0.67°°°**	**0.85°°°**
FatCSA								−040**	0.11	0.13	- 0.19*	- 0.11	- 0.14
								**−0.48^^**	−0.14	**0.38^^**	−0.05	−0.18	−0.16
								0.19	**0.37°°**	**0.39°°**	**−0.22°°**	**−0.43°°**	**−0.31°°**
SSIp									0.25*	0.52***	0.02	0.35**	0.56***
									**0.21^**	**0.53^^^**	0.11	**0.31^^**	**0.51^^^**
									**0.24°**	**0.69°°°**	0.16	**0.53°°°**	**0.67°°°**
BMAD										0.38***	- 0.01	0.16	0.02
										**0.40^^**	**0.21^**	**0.28^**	0.19
										**0.29°**	**0.39°°**	**0.57°°°**	**0.68°°°**
BMD											0.31**	0.78***	0.87***
											**0.28^**	**0.76^^^**	**0.72^^^**
											**0.71°°**	**0.67°°°**	**0.86°°°**
QUS *z*-score												0.56***	0.68***
												**0.51^^^**	**0.59^^^**
												**0.54°°°**	**0.65°°°**
AD-SoS													0.73***
													**0.68^^^**
													**0.86°°°**

The first line of each cell describes the correlation between bone parameters during transversal study of JIA patients; the second line of each cell describes the correlation between bone parameters during longitudinal study of JIA patients; the third line of each cell describes the correlation between bone parameters during transversal study of controls.

*JIA transversal study: *P < 0.05; **P < 0.005; ***P < 0.0005; JIA longitudinal study: ^*P* < 0.05; ^^*P* < 0.005; ^^^*P* < 0.0005; °Controls transversal study: °*P* < 0.05; °°*P* < 0.005; °°°*P* < 0.0005. AD-SoS, amplitude-dependent speed of sound; BTT, bone transmission time. CBA, cortical bone area; CrtBMD, cortical bone mineral density; FatCSA, fat cross-sectional area; JIA, juvenile idiopathic arthritis; a; MuscleCSA, muscle cross-sectional area; SSIp, density-weighted polar section modulus; QUS, quantitative ultrasonography; Tot density, total density. TrbBMD, trabecular bone mineral density.

**Table 3:** Correlation coefficients (rho) between the bone traits and JIA characteristics in the JIA cohort and controls

**Bone traits**	**JIA phenotypes**									
	**Type at onset**	**Age at onset**	**Disease duration**	**Disease activity**	**Flares**	**Chronologic age**	**MTX**	**Systemic corticosteroids**	**Intraarticular corticosteroids**	**Biological**
TrbBMD	- 0.50**	−0.31**	-0.24**	−0.34**	−0.31**	0.05	0.28*	−0.56**	−0.48**	0.48**
	**−0.39^^**	**−0.39^^**	**−0.43^^**	**−0.45^^**	**−0.29^**	0.19	**0.32^^**	**−0.61^^**	**−0.52^^**	**0.39^^**
	-	-	-	-	-	**0.74°°°**	-	-	-	-
CrtBMD	0.08	0.32**	0.24*	−0.31**	−0.26*	0.64***	−0.28**	0.20	0.26*	0.36**
	0.16	**0.37^^**	**0.28^**	**−0.28^^**	**−0.34^^**	**0.68^^^**	**−0.31^^**	**0.24^**	**0.24^**	**0.44^^**
	-	-	-	-	-	**0.89°°°**	-	-	-	-
Tot density	−0.11	−0.14	- 0.40***	- 0.44***	- 0.38***	0.28*	−0.24*	−0.29*	−0.26*	0.32**
	**−0.25^**	−0.18	**−0.38^^**	**−0.36^^**	**−0.42^^**	**0.36^^**	**−0.29^**	**−0.34^^**	**−0.28^**	**0.46^^**
	-	-	-	-	-	**0.62°°**	-	-	-	-
CBA	0.08	−0.04	−0.18	−0.27*	−0.20*	0.46**	−0.22*	0.06	0.16	0.39**
	0.18	−0.16	**−0.21^**	**−0.31^^**	**−0.24^**	**0.54^^**	**−0.31^^**	0.14	0.14	**0.28^**
	-	-	-	-	-	**0.79°°°**	-	-	-	-
Bone area	−0.09	−0.12	−0.23*	−0.28*	−0.30*	0.09	−0.16	−0.16	−0.14	0.33**
	−0.12	−0.19	**−0.27^**	**−0.21^**	**−0.34^^**	0.10	−0.14	**−0.28^**	**−0.34^^**	**0.49^^**
	-	-	-	-	-	**0.73°°°**	-	-	-	-
MuscleCSA	−0.26**	−0.18	−0.16	−0.41**	−0.33**	0.35**	−0.17	−0.46**	−0.51***	0.26*
	**−0.29^^**	**−0.24^**	**−0.25^**	**−0.46^^**	**−0.38^^**	**0.40^^**	**−0.08**	**−0.54^^^**	**−0.56^^^**	**0.38^^**
	-	-	-	-	-	**0.73°°°**	-	-	-	-
FatCSA	31.9**	0.34**	0.18	0.35*	0.21*	0.04	0.26*	0.42**	0.26*	−0.19
	**36.6^^**	**0.39^^**	**0.29^**	**0.28^**	**0.27^**	0.13	**0.39^^**	**0.39^^**	**0.84^^**	**−0.31^^**
	-	-	-	-	-	**0.23°**	-	-	-	-
SSIp	0.03	0.09	0.56***	0.45**	0.38**	0.21*	0.36**	0.51***	0..46**	−0.29*
	0.17	0.19	**0.48^^**	**0.49^^**	**0.32^^**	**0.23^**	**0.34^^**	**0.64^^^**	**0.39^^**	**−0.33^^**
	-	-	-	-	-	**−0.25°**	-	-	-	-
BMAD	- 0.25*	0.48***	−0.34**	−0.46***	−0.38**	0.38**	- 0.39**	- 0.45**	- 0.40**	0.43**
	**−0.23^**	**0.52^^^**	**−0.42^^**	**−0.51^^^**	**−0.34^^**	**0.47^^**	**−0,41^^**	**--0.49^^**	**−0.43^^**	**0.51^^^**
	-	-	-	-	-	**0.79°°°**	-	-	-	-
BMD	0.16	0.34**	−0.27*	−0.36**	−0.27*	0.38**	−0.36**	−0.53***	−0.41**	0.29*
	0.19	**0.36^^**	**−0.32^^**	**−0.39^^**	**−0.31^^**	**0.58^^**	**−0.44^^**	**−0.59^^^**	**−0.46^^**	**0.39^^**
	-	-	-	-	-	**0.88°°°**	-	-	-	-
QUS *z*-score	- 0.25*	−0.27*	−0.35**	−0.41**	−0.22*	0.11	0.16	−0.38**	−0.36**	0.21*
	**−0.21^**	**−0.33^^**	**−0.39^^**	**−0.33^^**	**−0.27^**	**−0.10**	0.12	**−0.46^^**	**−0.44^^**	**0.33^^**
	-	-	-	-	-	0.17	-	-	-	-
AD-SoS	−0.18	−0.37**	−0.51***	−0.39**	−0.47**	0.69***	−0.24*	−0.59***	−0.57***	0.61***
	**−0.23^**	**−0.42^^**	**−0.46^^**	**−0.31^^**	**−0.32^^**	**0.58^^^**	−0.38^^	**−0.65^^^**	**−0.51^^^**	**0.51^^^**
	-	-	-	-	-	**0.71°°°**	-	-	-	-
BTT	−0.57***	−0.51***	−0.67***	−0.47**	−0.27*	0.43**	−0.36*	−0.61***	−0.65***	0.61***
	**−0.62^^^**	**−0.64^^^**	**−0.52^^^**	**−0.43^^**	**−0.32^^**	**0.66^^**	**−0.48^^**	**−0.72^^^**	**−0.78^^^**	**0.67^^^**
	-	-	-	-	-	**0.84°°°°**	-	-	-	-

The first line of each cell describes the correlation between bone parameters and JIA phenotypes during transversal study of JIA patients; the second line of each cell describes the correlation between bone parameters and JIA phenotypes during longitudinal study of JIA patients; the third line of each cell describes the correlation between bone parameters during transversal study of controls.

JIA transversal study: *P < 0.05; **P < 0.005; ***P < 0.0005; JIA longitudinal study: ^*P* < 0.05; ^^*P* < 0.005; ^^^*P* < 0.0005; Controls transversal study: °*P* < 0.05; °°*P* < 0.005; °°°*P* < 0.0005.

AD-SoS, amplitude-dependent speed of sound; BMAD, bone mineral apparent density; BMD, bone mineral density; BTT, bone transmission time; CBA, cortical bone area; CrtBMD, cortical bone mineral density; FatCSA, fat cross-sectional area; JIA, juvenile idiopathic arthritis; MTX, methotrexate; MuscleCSA, muscle cross-sectional area; QUS, quantitative ultrasonography; SSIp, density-weighted polar section modulus; TrbBMD, trabecular bone mineral density; Tot density, total density.

### Longitudinal evaluation

At the time of the second evaluation, the median chronologic age was 17.7 years (range, 10.2 to 29.6 years). At this time, disease duration was 7.2 ± 4.3 years, and mean disease activity, 2.8 years. Disease duration was not significantly different among the subgroups of patients (Table 1). Besides, at longitudinal evaluation, 42.2% of the JIA patients had active disease, whereas 57.8% of patients had remission/inactive disease.

**Figure 2:**
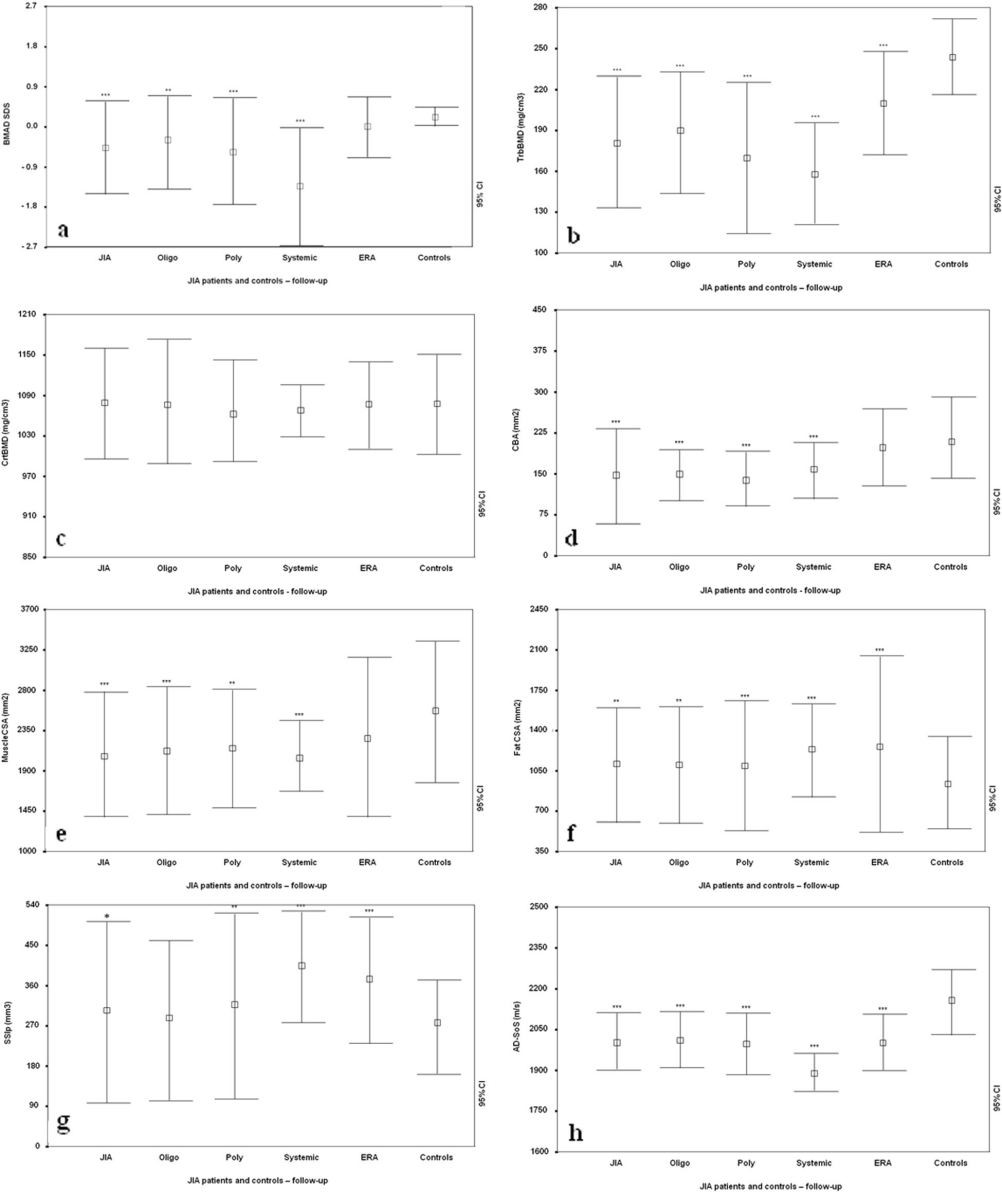
**Longitudinal evaluation.** Spine bone mineral apparent density (spine BMAD) SDS **(a),** trabecular bone mineral density (TrabBMD) **(b),** cortical bone mineral density (CrtBMD) **(c),** cortical bone area (CBA) **(d),** muscle cross-sectional area (MuscleCSA) **(e),** fat cross-sectional area (FatCSA) **(f),** density-weighted polar section modulus [SSIp] **(g),** and Ad-SoS **(h)** in patients with oligoarticular, polyarticular, systemic, and enthesitis-arthritis (ERA) JIA versus controls. Bars represent means and 95% confidence intervals. **P < 0.005; ***P < 0.001.

The longitudinal evaluation confirmed that the JIA patients had a reduced spine BMAD SDS in comparison with controls (−0.49 ± 1.10 versus 0.13 ± 0.32; *P* < 0.001). These results were still observed when the subjects were divided into oligoarticular (−0.29 ± 1.13; *P* < 0.005), polyarticular (−0.55 ± 1.24; *P* < 0.0001), and systemic subtypes (−1.36 ± 1.33; *P* < 0.0001), but not in the ERA subtype (0.05 ± 0.71; *P* = NS) (Figure 2a). Evaluating the differences among the JIA subgroups, oligoarticular (−0.36 ± 0.80 versus -0.29 ± 1.11), polyarticular (−0.73 ± 1.01 versus -0.55 ± 1.23), and ERA (0.01 ± 0.52 versus 0.05 ± 0.71; *P* = NS); no differences with respect to the findings in the first evaluation were noted, except for patients with systemic onset (−1.34 ± 1.20 versus -1.46 ± 1.44; *P* < 0.0001).

The JIA patients were confirmed to have significantly lower levels of TrbBMD (Figure 2b) than controls (181.9 ± 48.8 versus 244.9 ± 28.5 mg/cm^3^, *P* < 0.0001). These results were observed also in the oligoarticular (190.6 ± 44.7, *P* < 0.0001), polyarticular (169.9 ± 46.4, *P* < 0.0001), systemic (158.1 ± 38.1, *P* < 0.0001), and ERA subtypes (210.1 ± 37.4, *P* < 0.0001). With respect to the first examination, we did not find any significant differences among the JIA patients (181.9 ± 48.8 versus 179.5 ± 58.6 mg/cm^3^, *P* = NS), even when they were divided into oligoarticular (190.6 ± 44.7 versus 191.9 ± 43.2 mg/cm^3^), ERA (210.1 ± 37.4 versus 209.6 ± 44.4 mg/cm^3^), polyarticular (169.9 ± 46.4 versus 172.5 ± 55.5 mg/cm^3^), and systemic-onset (158.1 ± 38.1 versus 157.6 ± 39.3 mg/cm^3^) subtypes. Conversely, the controls showed a significant increase of TrbBMD with age (244.9 ± 28.5 versus 217.7 ± 26.3 mg/cm^3^, *P* < 0.0001).

The JIA patients did not show any significant differences in CrtBMD (Figure 2c) with respect to controls (1,079.8 ± 82.1 versus 1,078.3 ± 73.1, *P* = NS), even when differentiated by subtype (oligoarticular: 1,076.7 ± 86.0; polyarticular: 1,066.7 ± 80.6; ERA: 1,077.1 ± 63.2; systemic onset: 1,068.9 ± 39.6 mg/cm^3^, *P* < 0.0001). In comparison to the first examination, a significant increase in CrtBMD for the JIA patients (1,079.8 ± 82.1 versus 1,050.2 ± 126.5 mg/cm^3^, *P* < 0.05) was observed, although no differences among the oligoarticular (1,076.7 ± 86.0 versus 1,057.5 ± 142.4 mg/cm^3^), polyarticular (1,066.7 ± 80.6 versus 1,068.1 ± 76.8 mg/cm^3^), and ERA (1,077.1 ± 63.2 versus 1,070.4 ± 71.2 mg/cm^3^) subgroups were detected; a significant difference was noted for the systemic-onset subgroup (1,068.9 ± 39.6 versus. 994.0 ± 87.3 mg/cm^3^, *P* < 0.0001). The controls showed a significant increase of CrtBMD with age (1,078.3 ± 73.1 versus 1,039.6 ± 79.3, *P* < 0.05).

However, the JIA patients’ CBA values (Figure 2d) were confirmed to be significantly reduced with respect to the controls (149.0 ± 87.6 vs*.* 214.3 ± 74.3 mm^2^, *P* < 0.0001); these results were also observed when the subjects were divided into oligoarticular (149.8 ± 45.0 mm^2^; *P* < 0.0001), polyarticular- (139.46 ± 47.50 mm^2^, *P* < 0.0001), and systemic-onset subgroups (160.5 ± 49.5 mm^2^; *P* < 0.005), but not for the ERA subtype (198.5 ± 69.2 mm^2^; *P* = NS). With respect to the first evaluation, we did not observe significant differences across all JIA patients (149.0 ± 87.6 versus 136.3 ± 122.1 mm^2^, *P* = NS), nor did we observe such differences for the oligoarticular (149.8 ± 45.0 versus 148.5 ± 112.0 mm^2^; *P* = NS), ERA (198.5 ± 69.2 versus 187.5 ± 64.5 mm^2^, *P* = NS), and polyarticular (186.3 ± 131.1 versus 139.5 ± 47.5 mm^2^, *P* = NS) subgroups; however, the systemic-onset patients (160.5 ± 49.5 versus 126.3 ± 49.0 mm^2^; *P* < 0.05) had significantly increased values.

Muscle CSA (Figure 2e) was confirmed to be significantly reduced in JIA with respect to controls (2,083.7 ± 697.8 versus 2579.3 ± 794.5 mm^2^, *P* < 0.0001). When the patients were divided into subgroups, we observed a significant difference in the oligoarticular (2,126.5 ± 711.9, *P* < 0.0001), polyarticular (2,146.7 ± 670.7 mm^2^, *P* < 0.001), and systemic (2,056.9 ± 401.0 mm^2^, *P* < 0.0001) subtypes but not in the ERA subtype (2,281.2 ± 887.9 mm^2^, *P* = NS). In comparison to the first examination, we did not observe significant differences in the whole group of JIA patients (2,112.7 ± 732.4 versus 2,083.7 ± 697.8 mm^2^, *P* = NS) nor in the oligoarticular (2,126.5 ± 711.9 versus 2,167.7 ± 870, *P* = NS), polyarticular (2,146.7 ± 670.7 versus 2,100.5 ± 764.4 mm^2^, *P* = NS), systemic-onset (2,056.9 ± 401.0 versus 2,023.2 ± 632.8 mm^2^, *P* = NS), and ERA (2,420.2 ± 973.1 mm^2^, *P* = NS) subgroups. Conversely, for the controls, we observed a significant increase with age (2,579.3 ± 794.5 versus 2,398.5 ± 773.3 mm^2^, *P* < 0.001).

Fat CSA (Figure 2f) was still significantly increased in the JIA patients compared with controls (1,110.1 ± 483.7 versus 945.8 ± 394.2 mm^2^, *P* < 0.005), a finding that was similar for all subtypes (oligoarticular: 1,103.2 ± 489.3 mm^2^, *P* < 0.005; polyarticular: 1,099.0 ± 565.2 mm^2^, *P* < 0.0001; systemic: 1,255.5 ± 423.1 mm^2^, *P* < 0.0001; ERA: 1,289.7 ± 766.8 mm^2^, *P* < 0.0001). With respect to the first examination, we did not find any significant difference for the whole group of JIA patients (1,110.1 ± 483.7 versus 1,143.1 ± 649.1 mm^2^, *P* = NS) nor for any subtype (oligoarticular: 1,103.2 ± 489.3 versus 1,129.6 ± 451.0, *P* = NS; polyarticular: 1,099.0 ± 565.2 versus 1,080.9 ± 504.8 mm^2^, *P* = NS; systemic onset: 1,255.5 ± 423.1 versus 1,139.7 ± 351.9 mm^2^, *P* = NS; ERA: 1,289.7 ± 766.8 versus 1,335.2 ± 748.2 mm^2^, *P* = NS).

The SSIp longitudinal evaluation (Figure 2g) showed that the JIA patients had slightly significant lower levels than the controls (306.8 ± 200.7 versus 276.7 ± 110.4 mm^3^, *P* < 0.05), which was also found in the oligoarticular (287.6 ± 178.4, *P* = NS) and polyarticular subsets (316.1 ± 209.4, *P* < 0.005). In contrast, the subjects with systemic onset (410.4 ± 132.1, *P* < 0.001) and ERA (374.1 ± 139.1, *P* < 0.001) had SSIp values significantly increased above those of the controls (Figure 2). At follow-up, we detected significant differences in the JIA patients (306.8 ± 250.7 versus 246.3 ± 140.2 mm^3^, *P* < 0.05), but not in the controls (276.7 ± 110.4 versus 284.0 ± 127.9 mm^3^, *P* = NS). The results were replicated when JIA patients were divided into oligoarticular (251.7 ± 144.5 versus 287.6 ± 178.4 mm^3^, *P* = NS) and ERA (374.1 ± 139.1 versus 403.1 ± 189.9 mm^3^, *P* = NS) subgroups, whereas SSIp was significantly increased in the polyarticular- (314.1 ± 329.4 versus 220.2 ± 82.9 mm^3^, *P* < 0.05) and systemic-onset (400.4 ± 157.1 versus 228.0 ± 68.6 mm^3^, *P* < 0.001) subgroups.

Finally, longitudinal follow-up showed that AD-SoS (Figure 2h) remained significantly reduced (2,003.0 ± 101.1 versus 2152.8 ± 118.9 m/s, *P* < 0.001) in all subtypes of the JIA patients (oligoarticular: 2,016.1 ± 95.3 m/s; polyarticular: 1,993.3 ± 108.0 m/s; systemic: 1,897.0 ± 67.7 m/s; ERA: 2,000.4 ± 100.1 m/s, *P* < 0.0001). With respect to the first examination, we did not find significant differences for any JIA subgroups. The results remained comparable when we evaluated QUS *z*-scores between the JIA patients and controls (−0.5 ± 1.5 versus 0.2 ± 1.8, *P* < 0.005) and between the subtypes and controls (polyarticular: −0.7 ± 1.5; systemic: −3.3 ± 1.0; ERA: −0.7 ± 1.4, *P* < 0.0001); this finding was not replicated for the oligoarticular-onset subgroup (−0.2 ± 1.3, *P* = NS). Analyzing the first examination, we did not find significant differences for any subset of JIA patients.

The correlations among the variables in the longitudinal study are reported in Tables 2 and 3.

## Discussion

This study confirms our previously published data and further emphasizes that patients with JIA, although currently effective therapies are available, still manifest impaired bone mass and architecture. In addition, these patients seem not to reach a normal condition over time, potentially having a high risk of osteoporosis in early adulthood [3]. This characteristic is especially notable for children with polyarticular or systemic onset, whereas those with the oligoarticular and ERA subtypes do not show low values of BMAD SDS and other bone-density and quality determinants.

Furthermore, our results show remarkable evidence for significant musculoskeletal deficits in JIA patients and persistent impaired bone accrual over a period of years, thereby leading to further reductions of bone mass, in agreement with previously reported data [1,30,31]. Our baseline results using pQCT elucidate substantial muscle-skeletal deficits, partially due to the underlying inflammation typical of JIA. Nevertheless, our longitudinal data show that this pattern likely represents the combined effects of decreased biomechanical loading by muscle forces and increased inflammatory cytokines, because only patients with systemic onset experienced significant amelioration from antiinflammatory treatments.

Indeed, as muscles increase during growth, bones adjust by increasing in their dimensions and strength. The capacity of bone to respond to mechanical loading with increased bone strength is at its maximum during childhood [1]. Numerous studies have demonstrated that physical activity during childhood promotes cortical bone acquisition, either because of greater periosteal expansion or greater endosteal contraction [1,30].

In the last decades, new antirheumatic drugs, such as methotrexate and anti-TNF-α agents, have radically altered the course and outcome of JIA [32]. Despite these changes, numerous studies have documented persistent impaired bone mass and density in the majority of JIA patients [1,3]. This may suggest that current therapies, despite improving overall skeletal health, do not support adequate bone mass with respect to controls across nearly all JIA subgroups, thereby causing subsequent osteopenia and osteoporosis [3]. This hypothesis may be confirmed by the fact that many patients in our study did not have active arthritis at the time of evaluation.

Thus, bone alterations in JIA likely represent a mixed defect of bone development and lower muscle strength [6]. Our data show that young adults with a history of JIA may be susceptible to deleterious alterations in cortical bone strength and trabecular bone density, placing them at greater risk of fracture in the future. To reduce the risk, close monitoring of BMD, better control of disease activity, dietary intake of calcium and vitamin D, and especially greater physical activity may be advocated to support greater bone mass [3]. Thus, interventions aimed to improve muscle mass and strength may optimize bone health, resulting in a better PBM.

However, our data provide evidence that children and adolescents with ERA seem not to have significant reductions in bone mass with respect to controls, although these patients may have lower levels of physical activity, poorer physical health, and more bodily pain compared with oligoarticular or polyarticular subtypes of patients and controls [33]. So reduced physical activity and reduction of muscular mass may not completely explain these differences between JIA patients and controls and between patients with ERA and other JIA subgroups. It is possible that the reduced physical activity may be critical in conditioning the missed or reduced bone accrual, whereas the reduction of BMAD, which is frequently present at diagnosis, may be more related to inflammation. Limited data are available in the literature regarding bone status in patients with ERA. Burnham *et al.*[6], by using pQCT of the left tibia, observed reduced trabecular vBMD *z*-scores in this subtype of JIA.

The observation that loss of bone mass in ankylosing spondylitis (AS) seems to occur only in patients with persistent active disease, strongly suggests that the inflammatory activity of the disease itself plays a major role in the pathophysiology of the early bone mineral disorders observed in these patients [34]. Disease duration may also have a role in contributing to impaired BMD in these patients.

Another concern of our study is the high values of fatCSA detected in the JIA patients with respect to controls. The link between fat area and BMD remains unclear. However, recent studies led researchers to hypothesize a strong relation among leptin, metabolic state, and immunologic self-tolerance [35]. Moreover, chronic systemic inflammation, present in chronic arthritis, has been shown to increase lipogenesis in nonadipose tissues and lipolysis in white adipose tissue, resulting in ectopic lipid deposition in nonadipose tissues, such as muscle and liver [36]. The production of proinflammatory cytokines, characterizing an autoimmune disorder such as SLE, could be associated with an increased secretion of leptin and ectopic lipid accumulation in skeletal muscles, and, conversely, with higher rates of bone turnover and consequent loss of bone mass.

Our data also suggest that JIA patients, except for those with oligoarticular onset, do not experience improvements in their QUS *z-*scores with respect to baseline. It has been postulated that QUS may reflect qualitative aspects of bone, such as elasticity and microarchitectural characteristics [37] and may potentially be suitable for measuring several of the various determinants of bone strength [38]. Therefore, although during the follow-up, DXA may show stable values, the other methods may indicate the contrary: a decrease of bone parameters in some JIA subgroups, thus better reflecting the evaluation of skeletal status.

## Conclusions

Children with JIA are at risk for deleterious alterations in cortical bone strength and trabecular bone density, and consequently, for fractures. In contrast to other studies, our study demonstrated that the pronounced bone deficits in JIA are possibly also related to reductions in muscle cross-sectional area. Thus, bone alterations in JIA likely represent a mixed defect of bone remodeling and, in some cases, lower muscle forces. Future clinical trials assessing bone-active therapies and mechanical-loading interventions are required.

## Abbreviations

AD-SoS: Amplitude-dependent speed of sound; BMAD: bone mineral apparent density; BMD: bone mineral density; BMI: body mass index; BTT: bone transmission time; CBA: cortical bone area; C-HAQ: Childhood Health Assessment Questionnaire; CrtBMD: cortical bone mineral density; DXA: dual-energy x-ray absorptiometry; ERA: enthesitis-related arthritis; FatCSA: fat cross-sectional area; JIA: juvenile idiopathic arthritis; MuscleCSA: muscle cross-sectional area; NSAIDs: nonsteroidal antiinflammatory drugs; PBM: peak bone mass; pQCT: peripheral quantitative computed tomography; QUS: quantitative ultrasonography; SDS: standard deviation score; SSIp: density-weighted polar-section modulus; TotBMD: total bone mineral density; TrbBMD: trabecular bone mineral density; vBMD: volumetric BMD.

## Competing interests

The authors declare that no conflicts of interest could be perceived as prejudicing the impartiality of the research reported.

## Authors’ contributions

SS: conception and design, endocrinologic evaluation, manuscript writing, and final approval of the manuscript. LC: conception and design, data collection and analysis, manuscript writing, and final approval of the manuscript. FB: rheumatologic evaluation, data collection and analysis, manuscript writing, and final approval of the manuscript. CS: data collection and analysis, critical revision, and final approval of the manuscript. MMC: rheumatologic evaluation, critical revision, and final approval of the manuscript. MLB: endocrinologic evaluation, critical revision, and final approval of the manuscript. FF: rheumatologic evaluation, data collection, manuscript writing, critical revision, and final approval of the manuscript. All authors read and approved the final manuscript.

## References

[B1] Roth J Bechtold S Borte G Dressler F Girschick HJ Borte M Osteoporosis in juvenile idiopathic arthritis: a practical approach to diagnosis and therapy Eur J Pediatr 2007 166 775 784 10.1007/s00431-007-0484-1 17436015

[B2] Pepmueller PH Cassidy JT Allen SH Hillman LS Bone mineralization and bone mineral metabolism in children with juvenile rheumatoid arthritis Arthritis Rheum 1996 39 746 757 10.1002/art.1780390506 8639171

[B3] Stagi S Masi L Capannini S Cimaz R Tonini G Matucci-Cerinic M de Martino M Falcini F Cross-sectional and longitudinal evaluation of bone mass in children and young adults with juvenile idiopathic arthritis: the role of bone mass determinants in a large cohort of patients J Rheumatol 2010 37 1935 1943 10.3899/jrheum.091241 20516020

[B4] Gravallese EM Bone destruction in arthritis Ann Rheum Dis 2002 61 84 86 10.1136/ard.61.suppl_2.ii84 PMC1766721 12379632

[B5] Strand V Kavanaugh AF The role of interleukin-1 in bone resorption in rheumatoid arthritis Rheumatology (Oxford) 2004 43 10 16 10.1093/rheumatology/keh202 15150427

[B6] Burnham JM Shults J Dubner SE Sembhi H Zemel BS Leonard MB Bone density, structure, and strength in juvenile idiopathic arthritis: importance of disease severity and muscle deficits Arthritis Rheum 2008 58 2518 2527 10.1002/art.23683 18668565 PMC2705769

[B7] Cleary AG Lancaster GA Annan F Sills JA Davidson JE Nutritional impairment in juvenile idiopathic arthritis Rheumatology (Oxford) 2004 43 1569 1573 10.1093/rheumatology/keh387 15466896

[B8] Roth J Linge M Tzaribachev N Schweizer R Kuemmerle-Deschner J Musculoskeletal abnormalities in juvenile idiopathic arthritis: a 4-year longitudinal study Rheumatology (Oxford) 2007 46 1180 1184 10.1093/rheumatology/kem100 17500076

[B9] Häkkinen A Sokka T Kautiainen H Kotaniemi A Hannonen P Sustained maintenance of exercise induced muscle strength gains and normal bone mineral density in patients with early rheumatoid arthritis: a 5 year follow up Ann Rheum Dis 2004 63 910 916 10.1136/ard.2003.013003 15249317 PMC1755099

[B10] Falcini F Trapani S Civinini R Capone A Ermini M Bartolozzi G The primary role of steroids on the osteoporosis in juvenile rheumatoid arthritis patients evaluated by dual energy X-ray absorptiometry J Endocrinol Invest 1996 19 165 169 10.1007/BF03349860 8743282

[B11] Celiker R Bal S Bakkaloğlu A Ozaydin E Coskun T Cetin A Dinçer F Factors playing a role in the development of decreased bone mineral density in juvenile chronic arthritis Rheumatol Int 2003 23 127 129 12739043 10.1007/s00296-002-0265-0

[B12] Bonjour JP Chevalley T Ferrari S Rizzoli R The importance and relevance of peak bone mass in the prevalence of osteoporosis Salud Publica Mex 2009 51 S5 S17 19287894 10.1590/s0036-36342009000700004

[B13] Varonos S Ansell BM Reeve J Vertebral collapse in juvenile chronic arthritis: its relationship with glucocorticoid therapy Calcif Tissue Int 1987 41 75 78 3115548 10.1007/BF02555248

[B14] Henderson CJ Panush RS Diets, dietary supplements, and nutritional therapies in rheumatic diseases Rheum Dis Clin North Am 1999 25 937 968 ix 10.1016/S0889-857X(05)70112-5 10573768

[B15] Wey HE Binkley TL Beare TM Wey CL Specker BL Cross-sectional versus longitudinal associations of lean and fat mass with pQCT bone outcomes in children J Clin Endocrinol Metab 2011 96 106 114 10.1210/jc.2010-0889 20926531 PMC3038473

[B16] Baroncelli GI Federico G Vignolo M Valerio G del Puente A Maghnie M Baserga M Farello G Saggese G Phalangeal Quantitative Ultrasound Group Cross-sectional reference data for phalangeal quantitative ultrasound from early childhood to young-adulthood according to gender, age, skeletal growth, and pubertal development Bone 2006 39 159 173 10.1016/j.bone.2005.12.010 16473568

[B17] Falcini F Bindi G Ermini M Galluzzi F Poggi G Rossi S Masi L Cimaz R Brandi ML Comparison of quantitative calcaneal ultrasound and dual energy X-ray absorptiometry in the evaluation of osteoporotic risk in children with chronic rheumatic diseases Calcif Tissue Int 2000 67 19 23 10.1007/s00223001090 10908407

[B18] Górska A Urban M Bartnicka M Zelazowska-Rutkowska B Wysocka J Bone mineral metabolism in children with juvenile idiopathic arthritis: preliminary report Ortop Traumatol Rehabil 2008 10 54 62 18391906

[B19] Kotaniemi A Savolainen A Kröger H Kautiainen H Isomäki H Weight-bearing physical activity, calcium intake, systemic glucocorticoids, chronic inflammation, and body constitution as determinants of lumbar and femoral bone mineral in juvenile chronic arthritis Scand J Rheumatol 1999 28 19 26 10092160 10.1080/03009749950155733

[B20] Lurati A Cimaz R Gattinara M Gerloni V Teruzzi B Salmaso A Fantini F Skeletal mineralization in a prepubertal female population affected by juvenile idiopathic arthritis Reumatismo 2008 60 224 229 18854886

[B21] Pereira RM Corrente JE Chahade WH Yoshinari NH Evaluation by dual X-ray absorptiometry (DXA) of bone mineral density in children with juvenile chronic arthritis Clin Exp Rheumatol 1998 16 495 501 9706436

[B22] Lien G Selvaag AM Flatø B Haugen M Vinje O Sørskaar D Dale K Egeland T Førre Ø A two-year prospective controlled study of bone mass and bone turnover in children with early juvenile idiopathic arthritis Arthritis Rheum 2005 52 833 840 10.1002/art.20963 15751052

[B23] Petty RE Southwood TR Manners P Baum J Glass DN Goldenberg J He X Maldonado-Cocco J Orozco-Alcala J Prieur AM Suarez-Almazor ME Woo P International League of Associations for Rheumatology International League of Associations for Rheumatology classification of juvenile idiopathic arthritis: second revision, Edmonton, 2001 J Rheumatol 2004 31 390 392 14760812

[B24] Cacciari E Milani S Balsamo A Spada E Bona G Cavallo L Cerutti F Gargantini L Greggio N Tonini G Cicognani A Italian cross-sectional growth charts for height, weight and BMI (2 to 20 yr) J Endocrinol Invest 2006 29 581 593 10.1007/BF03344156 16957405

[B25] Tanner JM Whitehouse RH Clinical longitudinal standards for height, weight, height velocity, weight velocity, and stages of puberty Arch Dis Child 1976 51 170 179 10.1136/adc.51.3.170 952550 PMC1545912

[B26] Kröger H Kotaniemi A Vainio P Alhava E Bone densitometry of the spine and femur in children by dual-energy x-ray absorptiometry Bone Miner 1992 17 75 85 1581707 10.1016/0169-6009(92)90712-m

[B27] Kröger H Vainio P Nieminen J Kotaniemi A Comparison of different models for interpreting bone mineral density measurements using DXA and MRI technology Bone 1995 17 157 159 10.1016/S8756-3282(95)00162-X 8554924

[B28] D’Elia G Caracchini G Cavalli L Innocenti P Bone fragility and imaging techniques Clin Cases Miner Bone Metab 2009 6 234 246 22461252 PMC2811356

[B29] Brunner HI Lovell DJ Finck BK Giannini EH Preliminary definition of disease flare in juvenile rheumatoid arthritis J Rheumatol 2002 29 1058 1064 12022323

[B30] Roth J Palm C Scheunemann I Ranke MB Schweizer R Dannecker GE Musculoskeletal abnormalities of the forearm in patients with juvenile idiopathic arthritis relate mainly to bone geometry Arthritis Rheum 2004 50 1277 1285 10.1002/art.20128 15077312

[B31] Felin EM Prahalad S Askew EW Moyer-Mileur LJ Musculoskeletal abnormalities of the tibia in juvenile rheumatoid arthritis Arthritis Rheum 2007 56 984 994 10.1002/art.22420 17328076

[B32] Simonini G Giani T Stagi S de Martino M Falcini F Bone status over 1 yr of etanercept treatment in juvenile idiopathic arthritis Rheumatology (Oxford) 2005 44 777 780 10.1093/rheumatology/keh592 15741191

[B33] Flatø B Hoffmann-Vold AM Reiff A Førre Ø Lien G Vinje O Long-term outcome and prognostic factors in enthesitis-related arthritis: a case–control study Arthritis Rheum 2006 54 3573 3582 10.1002/art.22181 17075863

[B34] Gratacós J Collado A Pons F Osaba M Sanmartí R Roqué M Larrosa M Múñoz-Gómez J Significant loss of bone mass in patients with early, active ankylosing spondylitis: a followup study Arthritis Rheum 1999 42 2319 2324 10.1002/1529-0131(199911)42:11<2319::AID-ANR9>3.0.CO;2-G 10555026

[B35] Matarese G Procaccini C De Rosa V At the crossroad of T cells, adipose tissue, and diabetes Immunol Rev 2012 249 116 134 10.1111/j.1600-065X.2012.01154.x 22889219

[B36] Mei M Zhao L Li Q Chen Y Huang A Varghese Z Moorhead JF Zhang S Powis SH Li Q Ruan XZ Inflammatory stress exacerbates ectopic lipid deposition in C57BL/6 J mice Lipids Health Dis 2011 10 110 10.1186/1476-511X-10-110 21718499 PMC3146847

[B37] Glüer C Wu C Jergas M Goldstein S Genant H Three quantitative ultrasound parameters reflect bone structure Calcif Tissue Int 1994 55 45 52 10.1007/BF00310168 7922789

[B38] Faulkner KG Glüer CC Majumdar S Lang P Engelke K Genant HK Noninvasive measurements of bone mass, structure, and strength: current methods and experimental techniques AJR Am J Roentgenol 1991 157 1229 1237 10.2214/ajr.157.6.1950872 1950872

